# The Clinical Profile of Children With Hepatitis A Infection: An Observational Hospital-Based Study

**DOI:** 10.7759/cureus.28290

**Published:** 2022-08-23

**Authors:** Sharanya Murlidharan, Avinash L Sangle, Madhuri Engade, Ajay B Kale

**Affiliations:** 1 Pediatrics, Mahatma Gandhi Mission (MGM) Medical College and Hospital, Aurangabad, IND

**Keywords:** hepatitis a, waterborne disease, serum glutamate oxaloacetate transaminase, infectious causes of fever with jaundice, total serum bilirubin

## Abstract

Introduction: Hepatitis A is a frequent form of hepatitis, especially in children. The changing epidemiology of the disease signifies the need for descriptive data concerning the clinical presentation and outcome of hepatitis A in children. The present study describes the clinical and biochemical profile of children with hepatitis A infection from a tertiary care center in the Aurangabad district of Maharashtra in Western India.

Methods: One hundred patients between one and 18 years of age, presenting with symptoms/signs such as nausea, anorexia, vomiting, jaundice, abdominal pain, tender hepatomegaly, bleeding manifestations, or encephalopathy, were enrolled for the study. Serologically confirmed cases by detecting immunoglobulin M (IgM) antibodies against hepatitis A virus (HAV) were enrolled in the study. A detailed case proforma noted the clinical features and details such as age, gender, area, water supply, socioeconomic status, season, and biochemical parameters.

Results: Most patients (45%) were among the age group of one to five years. Fever was reported in 96 (96%) patients, abdominal pain in 78 (78%) patients, dark-colored urine in 65 (65%) patients, vomiting in 47 (47%) patients, and anorexia in 63 (63%) patients. Icterus was found in 80 (80%) patients and hepatomegaly in 74 (74%) patients. In 61 (61%) patients, serum total bilirubin level on the first day was 1-4 mg/dL. Sixty-five (65%) patients were using tap water as the water source, and the maximum number of patients (43%) came in August. Most patients belonged to the class IV group (61%) as per the modified Kuppuswamy classification.

Conclusions: Most patients were 10 years or below, presenting predominantly with fever, abdominal pain, dark-colored urine, vomiting, and anorexia. Icterus and hepatomegaly were found in three-fourths or more of the patients. Around monsoon (June to September), there was the highest frequency of cases, and the socioeconomic status of most of the patients was within lower or upper-lower categories.

## Introduction

Viral hepatitis has become a significant healthcare burden across India, along with other infectious diseases such as acquired immunodeficiency syndrome, malaria, and tuberculosis. Hepatitis A virus (HAV) is responsible for the most frequent form of acute viral hepatitis across the globe. Hepatitis A is mainly transmitted by the enteric route and is known to cause sporadic infections, as well as epidemics of acute viral hepatitis. A typical presentation of hepatitis A infection in symptomatic cases is a variable combination of nonspecific prodromal symptoms such as fever, weakness, malaise, nausea, anorexia, vomiting, and muscle and joint pains. With the onset of jaundice, there may be a decrease in these prodromal symptoms, but weakness, anorexia, and malaise may persist. Jaundice may last for several weeks. The peak infectivity precedes jaundice and the elevation of liver enzymes when there is a high concentration of HAV in the stools. After that, the infectivity declines with the appearance of jaundice [1-3]. In India, there is heterogeneity in the proportion of susceptible and exposed populations to HAV infection in various regions. A few decades ago, all the studied Indian newborns were found to have anti-HAV immunoglobulins, indicating 100% maternal antibody presence. However, recent literature reflects a fall in the prevalence, with 50%-60% of newborns reported to have anti-HAV immunoglobulins in cord blood [3].

Exposure to the HAV usually occurs early in life and is frequently subclinical, which may confer lifelong immunity. Very high exposure in childhood to the hepatitis A virus has been documented in India by various serological studies. There have been reports of change in the epidemiology of hepatitis A and the need for monitoring it, especially in resource-poor countries. The importance of serological surveys and disease surveillance for morbidity and mortality has been stressed to document changing epidemiology [4,5]. The present study presents the clinical, biochemical profile, and outcome of children with hepatitis A infection from a tertiary care center in Aurangabad district of Maharashtra province in Western India.

## Materials and methods

This descriptive observational study was conducted at Mahatma Gandhi Mission Medical College and Hospital, Aurangabad, from October 2018 to October 2020. The institutional ethics committee (letter number: MGM-ECRHS/2018/19) approved the study protocol. One hundred patients between one and 18 years of age attending the outpatient department or admitted to the hospital with clinical features suggestive of acute hepatitis such as nausea, anorexia, vomiting, jaundice, abdominal pain, tender and enlarged liver, bleeding manifestations, or encephalopathy; elevated transaminases or pediatric acute liver failure; and positive hepatitis A immunoglobulin M (IgM) tests were enrolled in the study. Patients with known cholestatic jaundice, biliary atresia, previously diagnosed chronic liver disease, and hepatotoxic drug intake were excluded. Informed consent of the parents/guardian was taken.

The children were clinically examined and evaluated for liver function tests (LFTs) and viral markers. A detailed case proforma included clinical features and details such as age, gender, area in terms of urban and rural areas with urban areas defined as those falling within the municipal corporation or municipality limits and rural areas as those outside these limits, water supply, socioeconomic status as per the modified Kuppuswamy classification 2019 (Appendix) [6], season, and biochemical parameters.

## Results

Most patients were in the age group of 1-5 years of age (45 (45%) cases), followed by 40 (40%) cases in the age group of 6-10 years (Table 1).

**Table 1:** Distribution of cases according to age

Age group (years)	Number of children
1-5	45
6-10	40
11-15	14
16-18	1
Total	100

There were 51 (51%) male and 49 (49%) female patients. Out of the 100 patients with hepatitis A infection, fever was reported in 96 (96%) patients, abdominal pain was reported in 78 (78%) patients, dark-colored urine was reported in 65 (65%) patients, vomiting was reported in 47 (47%) patients, and anorexia was reported in 63 (63%) patients.

In 32 (32%) and 33 (33%) patients, fever appeared on the second and the third day, respectively. In 14 (14%) patients, it came on the fourth day. In 18 (18%) patients, it came on the fifth day, whereas in three (3%) patients, it came on the first day.

Out of 100 patients with HAV, icterus was found in 80 (80%) patients, and hepatomegaly was found in 74 (74%) patients. In most of the patients (97 (97%)), the duration of illness was >5 days, and in only three (3%) patients, it was <5 days.

Table 2 reflects the total bilirubin levels on the day of presentation.

**Table 2:** Total bilirubin levels on the day of presentation

Serum total bilirubin level (mg/dL)	Number of cases on the day of presentation	
	Number of patients	Percentage (%)
0-4	61	61%
4.1-8	32	32%
8.1-12	6	6%
>12	1	1%
Total	100	100%

The serum total bilirubin level on the last day was between 0 and 4 mg/dL in 99 (99%) cases and between 8.1 and 12 mg/dL in only one (1%) patient. In the majority of patients (53 (53%)), the serum direct bilirubin level was found in the range of 1-4 mg/dL, followed by 43 (43%) patients in the range of 4.1-8 mg/dL. In three (3%) patients, the serum direct bilirubin level was in the range of 8.1-12 mg/dL, and in only one (1%) patient, the serum direct bilirubin level was found to be >12 mg/dL. The serum direct bilirubin level on the last day between 1 and 4 mg/dL was found in 99 (99%) cases and between 4.1 and 8 mg/dL was found in one (1%) patient.

Out of 100 patients with hepatitis A infection, in the majority of patients (55 (55%)), the serum indirect bilirubin level was found in the range of 0.6-1 mg/dL, followed by 36 (36%) patients in the range of 0-0.5 mg/dL. In four (4%) patients, the serum indirect bilirubin level was in the range of 1.1-1.5 mg/dL, and in only two (2%) patients, the serum indirect bilirubin level was found to be >2 mg/dL. The serum indirect bilirubin level on the last day was between 0 and 0.5 mg/dL in eight (8%) cases, between 0.6 and 1 mg/dL in 91 (91%) cases, and between 1.1 and 1.5 mg/dL in one (1%) case.

Table 3 shows the distribution of cases according to the serum glutamate oxaloacetate transaminase (SGOT) level on the day of presentation.

**Table 3:** Distribution of cases according to the SGOT level on the day of presentation SGOT: serum glutamate oxaloacetate transaminase The normal range of SGOT is 5-40 IU/dL.

SGOT level (IU/dL)	Number of cases on the day of presentation	
	Frequency	Percentage (%)
1-500	59	59%
501-1,000	28	28%
1,001-1,500	7	7%
1,501-2,000	1	1%
2,001-2,500	2	2%
2,501-3,000	1	1%
>3,000	2	2%
Total	100	100%

The SGOT level on the last day was between 1 and 500 IU/dL in 100 (100%) cases.

Table 4 shows the distribution of cases according to the SGPT level on the day of presentation.

**Table 4:** Distribution of cases according to the SGPT level on the day of presentation SGPT: serum glutamate pyruvate transaminase The normal range of SGOT is 7-56 IU/dL.

SGPT level (IU/dL)	Number of cases on the day of presentation	
	Frequency	Percentage (%)
1-500	63	63%
501-1,000	22	22%
1,001-1,500	6	6%
1,501-2,000	5	5%
2,001-2,500	2	2%
2,501-3,000	1	1%
>3,000	1	1%
Total	100	100%

Table 5 shows the distribution of cases according to ultrasound examination findings.

**Table 5:** Distribution of cases according to ultrasound examination findings

Physical findings	Number of cases	
	Frequency	Percentage (%)
Hepatomegaly	78	78%
Hepatomegaly with pseudo-thickening of the gallbladder wall	22	22%
Gallbladder wall thickening	22	22%
Splenomegaly	1	1%
Liver cirrhosis	2	2%
Ascites with pleural effusion	1	1%

Out of 100 patients with hepatitis A infection, 78 (78%) patients resided in rural areas, and 22 (22%) patients lived in urban areas. Out of 100 patients with hepatitis A infection, 65 (65%) patients were using tap water, whereas 35 (35%) patients were using well water as the main water supply.

Table 6 shows the distribution of cases according to complete blood test findings.

**Table 6:** Distribution of cases according to complete blood test findings The normal range of serum hemoglobin in children is 12 g/dL and above, the total leucocyte count is 4,000-11,000 mm^3^ of blood, and the platelet count is 1.5-4 lakh/mm^3^ of blood.

Serial number	Serum hemoglobin (g/dL)	Number of cases	Total leucocyte count/mm^3^ of blood	Number of cases	Platelet count (lakh/mm^3^)	Number of cases
1	≤8	1	≤4,000	3	≤1.5	0
2	8.1-10	0	4,000-6,000	94	1.6-2	0
3	10.1-12	27	6,001-8,000	3	2.1-2.5	72
4	12.1-14	70	8,001-10,000	0	2.6-3	3
5	≥14.1	2	≥10,001	0	≥3.1	25

Table 7 shows the distribution of cases according to seasonal variation.

**Table 7:** Distribution of cases according to seasonal variation

Serial number	Month of admission	Number of cases	Percentage (%)
1	January	5	5%
2	February	0	0%
3	March	6	6%
4	April	4	4%
5	May	0	0%
6	June	13	13%
7	July	0	0%
8	August	43	43%
9	September	9	9%
10	October	2	2%
11	November	18	18%
12	December	0	0%
Total		100	100%

Most of the patients belonged to the class IV group (61%) as per the modified Kuppuswamy classification, followed by class V (32%) and then class III (7%) (Table 8).

**Table 8:** Groups according to socioeconomic status as per the modified Kuppuswamy classification Class I: upper class, class II: upper-middle class, class III: lower-middle class, class IV: upper-lower class, class V: lower class

Modified Kuppuswamy classification	Number of cases
Class I	0
Class II	0
Class III	7
Class IV	61
Class V	32

The rate of fall of bilirubin/day was 1.15 mg/dL (standard deviation: 0.3541).

## Discussion

Our study results were in line with available studies from India. Kumar et al. have reported similar results in their study of 78 children with IgM anti-HAV-positive acute viral hepatitis. Fever and hepatomegaly were noted in most patients, with a greater than fivefold rise in transaminases in more than half of the patients [7]. A recent observational study from Uttar Pradesh, India, on 1,615 acute viral hepatitis patients during 2016-2017 concluded that hepatitis A was more common in childhood and that HAV IgM-seropositive cases were more prevalent in monsoon season [8]. Hepatitis A is common in children, and several other studies have reported the rainy season or contaminated water supply as frequently associated factors [9-13]. Jaundice, fatigue, and anorexia were the frequent symptoms, whereas icterus and hepatomegaly were the typical presenting signs in a study from South India [14]. Similar to our study results, the clinical presentation with jaundice, fever, vomiting, rise in serum bilirubin, and manifold rise in transaminases was reported by researchers from Chennai [15].

The limitations of the present study are that the study was a regional, hospital-based study and it has a smaller sample size, which limits generalizability. However, the study adds to the literature regarding the clinical profile of symptomatic patients attending a tertiary care hospital in Maharashtra in India with hepatitis A.

## Conclusions

The prevalence of hepatitis A infection was higher during the monsoon months (June to September) and among the children residing in rural areas or using tap water as the water source. The present study adds to the epidemiological data on hepatitis A from symptomatic patients attending a medical college and hospital in Maharashtra in India.

## Figures and Tables

**Figure 1:**
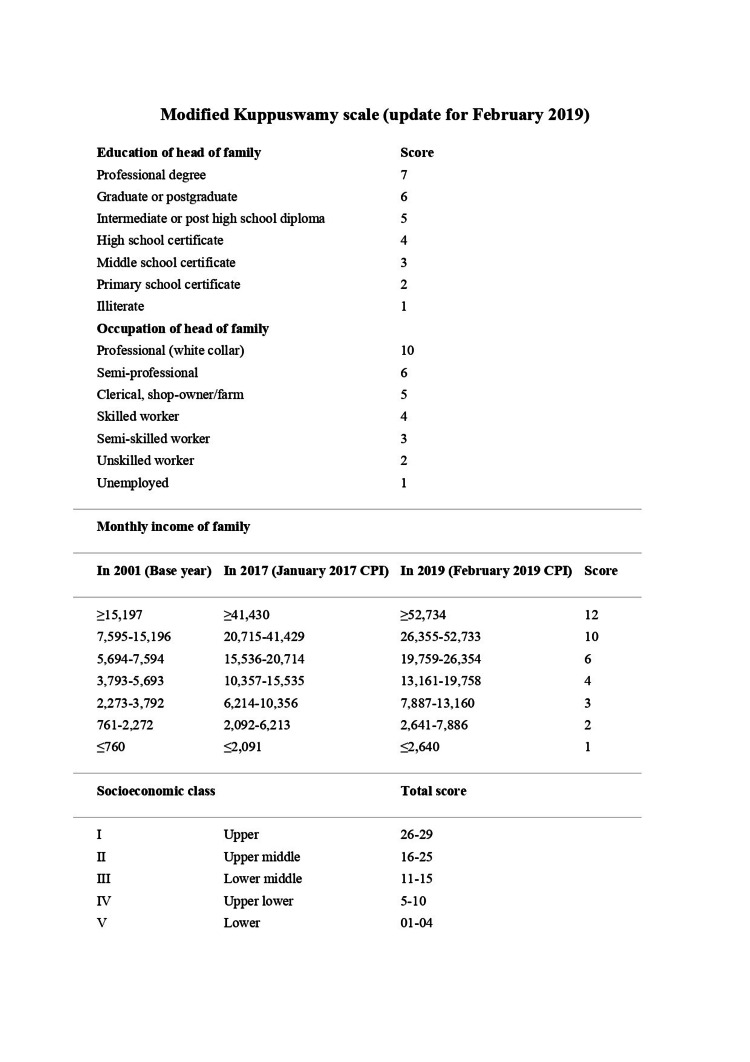
Modified Kuppuswamy classification

## Appendices

Figure 1 presents the socioeconomic status of the patients as per the modified Kuppuswamy classification (2019).
